# Association of Once-Daily MALDI-TOF MS Identification with Antibiotic Appropriateness and First-Modification Patterns in Emergency Department Bacteremia: A Retrospective Before–After Cohort Study †

**DOI:** 10.3390/antibiotics15040386

**Published:** 2026-04-10

**Authors:** Jack Yu-Shuo Lu, Yu-Hsun Wang, Shu-Ling Tzeng, Yuan-Ti Lee, Tzu-Chi Wu

**Affiliations:** 1 Institute of Medicine, Chung Shan Medical University, Taichung City 40201, Taiwan; 2 Department of Emergency Medicine, School of Medicine, Chung Shan Medical University, Taichung City 40201, Taiwan; 3 Department of Emergency Medicine, Chung Shan Medical University Hospital, Taichung City 40201, Taiwan; 4 Department of Medical Research, Chung Shan Medical University Hospital, Taichung City 40201, Taiwan; 5 School of Medicine, Chung Shan Medical University, Taichung City 40201, Taiwan; 6 Division of Infectious Diseases, Department of Internal Medicine, Chung Shan Medical University Hospital, Taichung City 40201, Taiwan; 7 Department of Emergency Medicine, Show Chwan Memorial Hospital, Changhua 50044, Taiwan; 8 Department of Post-Baccalaureate Medicine, College of Medicine, National Chung Hsing University, Taichung City 40227, Taiwan

**Keywords:** antibiotic, emergency department, bacteremia, MALDI-TOF MS, positive blood cultures

## Abstract

**Background:** Blood cultures are frequently obtained in the emergency department (ED), yet organism identification and subsequent antibiotic optimization commonly occur after hospital admission. Inappropriate empiric therapy remains common and is associated with adverse outcomes. MALDI-TOF MS can shorten the time to organism identification; however, real-world effectiveness may depend on laboratory cadence and stewardship support, and evidence for once-daily batch workflows without active antimicrobial stewardship is limited. **Method:** We performed a retrospective before–after cohort study at a tertiary medical center in central Taiwan, comparing positive blood cultures (PBCs) obtained in the ED before MALDI-TOF MS implementation (1 May–31 July 2018; conventional identification) and after implementation (1 September–30 November 2018; MALDI-TOF MS). Primary endpoints were appropriate antibiotic therapy at 24, 48, and 72 h after the first PBC report. Secondary endpoints included timing, location, and classification (escalation vs. de-escalation) of the first antibiotic modification. **Results:** After exclusions, 323 unique PBCs were analyzed (182 pre-implementation; 141 post-implementation). Baseline characteristics and clinical outcomes were similar, including in-hospital mortality (14.8% vs. 14.9%). Time to the initial positive report (Gram stain) and the final report (identification with antimicrobial susceptibility testing) did not differ significantly between periods. Appropriate antibiotic use at 24/48/72 h was comparable (75.3% vs. 76.6%, 82.4% vs. 80.1%, and 86.3% vs. 84.4%). The timing and pattern of the first antibiotic modification were also similar. In a secondary stratified analysis, patients modified before culture positivity had higher acuity and worse outcomes. **Conclusions:** Once-daily MALDI-TOF MS implementation was not associated with improved antibiotic appropriateness or modification patterns in ED bacteremia without active real-time stewardship oversight. Higher-frequency processing and real-time stewardship linkage may be required to translate faster diagnostics into timely therapeutic action.

## 1. Introduction

Infectious diseases with febrile presentations are among the most common chief complaints in the emergency department (ED), and progression to severe sepsis or septic shock is associated with mortality rates of 30–40% [1,2]. For ED patients with a bloodstream infection (BSI), about 15–30% do not receive appropriate empiric antibiotic (AEA) therapy, which has been associated with worse outcomes; resistance to empiric ED antibiotics has been reported to be as high as 14.5% [3,4]. Lower AEA rates have been observed in BSI caused by multidrug-resistant organisms, and inappropriate empiric therapy may even represent an independent predictor of 30-day mortality [5,6]. Moreover, delays in effective antibiotic therapy adversely affect sepsis outcomes; each hour’s delay in effective antibiotic therapy results in an estimated 7.6% decrease in survival [7,8].

Matrix-assisted laser desorption ionization time-of-flight mass spectrometry (MALDI-TOF MS) has been shown to significantly reduce time-to-identification and enable earlier AEA therapy, with recent meta-analyses reporting more frequent de-escalation and a shorter length of stay (LOS) [9,10,11]. Direct-from-positive-blood-culture MALDI-TOF MS and short-incubation protocols can reduce turnaround time by approximately one day in many laboratories, potentially increasing the proportion of patients receiving appropriate therapy within 24–48 h [12]. The most crucial distinction is that the combination of MALDI-TOF MS plus antimicrobial stewardship outperforms MALDI-TOF alone in moving patients to optimal/targeted therapy faster and in improving clinical outcomes, including mortality, in recent systematic reviews [13,14,15].

MALDI-TOF MS on isolated colonies growing on solid media is now a standard practice in high-income countries, and its effectiveness in ED-related workflows requires further demonstration of benefits in patient outcomes without real-time pharmacist or infectious diseases specialist supervision [1,13,14]. In addition, many studies have evaluated hospital-wide BSI cohorts in which a large fraction originates in the ED, making their findings ED-relevant even if not ED-exclusive [15]. ED-only MALDI-TOF MS studies remain uncommon. Nevertheless, multiple-drug-resistant pathogens in bacteremia have been sharply rising in East Asia, with limited treatment options and high mortality reported in multicenter studies; for example, vancomycin-resistant *Enterococcus faecium* and *Carbapenem-resistant Acinetobacter baumannii (CRAB)* bacteremia have been associated with mortality rates of up to 48% [16,17]. Despite the earlier organism identification achieved with MALDI-TOF MS, the prevalence of highly resistant pathogens remains a major challenge to ensuring appropriate empiric antibiotic therapy in real-world clinical practice.

Because blood cultures are frequently obtained in the ED but turn positive after patient disposition, the clinical impact of rapid organism identification depends on downstream inpatient workflows. We therefore evaluated whether implementing MALDI-TOF MS in a once-daily laboratory workflow, without real-time antimicrobial stewardship intervention, improves early antibiotic optimization in patients with positive blood cultures admitted in the ED. The primary outcome was the AEA prescribing rate during the early admission course among patients with PBCs. The secondary outcome was whether antibiotic changes among patients with PBCs were associated with differences in clinical outcomes.

## 2. Results

**Figure 1:**
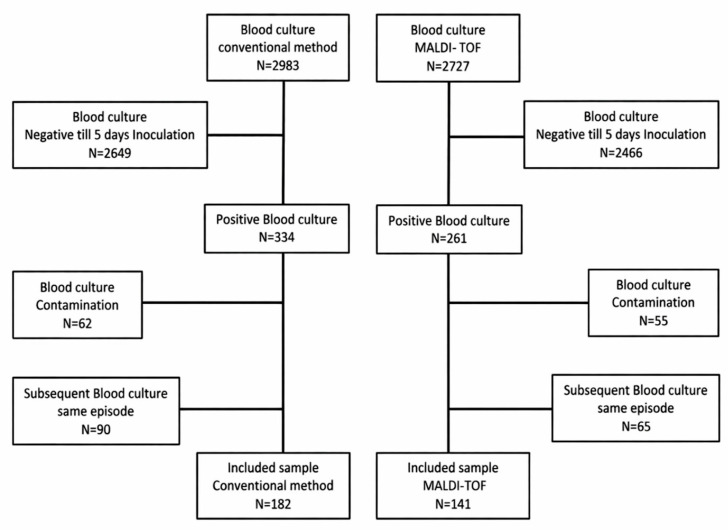
Flowchart of the inclusion process for blood cultures collected in the emergency department.

Between 1 May and 30 November 2018, we identified 343 positive blood cultures (PBCs); after excluding August 2018 (transition month), predefined contaminants, and duplicate blood cultures within the same infectious episode, 323 unique PBCs were analyzed: 182 in the pre-implementation (conventional identification; May 1–July 31) group and 141 in the post-implementation (MALDI-TOF MS; 1 September–30 November) group. During the study windows, 2983 and 2727 ED blood-culture sets were obtained in the pre- and post-periods, respectively; 2649 and 2446 sets were negative and excluded per protocol, with additional exclusions for predefined contaminants and duplicate isolates (Figure 1).

Baseline characteristics in the pre- and post-implementation PBC groups were similar with respect to age (mean 70.6 vs. 67.7 years, *p* = 0.06), male sex (47.8% vs. 50.4%, *p* = 0.655), Do Not Resuscitate (DNR)/hospice status (6.0% vs. 4.3%, *p* = 0.617), maintenance hemodialysis (4.9% vs. 7.8%, *p* = 0.354), and malignancy (24.7% vs. 30.5%, *p* = 0.259). Clinical outcomes showed no statistically significant differences in in-hospital mortality (14.8% vs. 14.9%, *p* = 0.988), ward length of stay (mean 11.1 vs. 13.5 days, *p* = 0.078), or ICU length of stay (mean 2.20 vs. 2.28 days, *p* = 0.92). Disposition to the ICU vs. the general ward did not differ between groups (*p* = 0.763) (Table 1).

Microbiology workflow times to the initial positive report (Gram stain) were comparable (mean 26.05 vs. 27.35 h; median 20 [IQR 16–26] vs. 21 [IQR 16–28] hours; *p* = 0.22). Final report times (organism identification with AST) were also similar (mean 81.46 vs. 82.46 h; median 69 [IQR 60–88] vs. 69 [IQR 61.5–86.5] hours, with no statistically significant difference (*p* = 0.81). Most PBCs were monomicrobial (89.6% vs. 89.4%). The leading pathogens were *Escherichia coli* (40.7% vs 38.3%), *Klebsiella pneumoniae*, and *Staphylococcus aureus* (ranked first to third in both periods). The fourth most frequent pathogens were methicillin-resistant *S. aureus* (MRSA, 3.8%) and *Pseudomonas aeruginosa* (5.7%). The distribution of presumed infection sources did not differ significantly (urinary tract 36.3% vs. 36.9%; gastrointestinal 23.1% vs. 23.4%; other 16.5% vs. 10.6%; *p* = 0.782).

Following the microbiology findings, we evaluated antibiotic changes and appropriateness. A higher proportion of patients in the post-implementation PBC group underwent escalation to broad-spectrum therapy within 24 h (19.2% vs. 24.8%, *p* = 0.407) and 48 h (7.1% vs. 10.6%, *p* = 0.345), though neither difference reached statistical significance. The primary outcomes—appropriate antibiotic use at 24, 48, and 72 h after the first PBC—were similar between groups: 75.3% vs. 76.6%, 82.4% vs. 80.1%, and 86.3% vs. 84.4%, respectively (Table 2.)

In the secondary outcome analysis, the pattern of the first antibiotic modification was comparable between groups: the mean time to first change was 25.46 vs. 31.16 h (*p* = 0.206); the proportion of PBCs that changed within ≤24 h was 36.8% vs. 35.5% and within 24–72 h was 22.0% vs. 22.7% (*p* = 0.316). More than half of the first changes represented broad-spectrum escalation (50.0% vs. 53.9%, *p* = 0.451), and the ICU was the most common location for the initial modification (75.7% vs. 92.0%); none of these contrasts achieved conventional thresholds for statistical detectability. Notably, 32.4% vs. 24.8% of patients had their antibiotics changed before any positive blood-culture signal, based instead on clinical judgment (*p* = 0.141), again showing no clear difference (Table 3).

We therefore conducted a secondary analysis stratified by the timing of the first change: before PBC (pre-PBC; N = 94) versus after PBC (post-PBC; N = 229). Baseline acuity differed between strata: septic shock was more frequent in the “pre-PBC” group (38.3% vs. 16.6%, *p* < 0.001) and qSOFA > 2 was likewise more frequent (22.3% vs. 8.3%, *p* = 0.001). Clinical outcomes were worse in this higher-acuity stratum: in-hospital mortality (21.3% vs. 12.2%, *p* = 0.04), total length of stay (mean 14.63 vs. 11.17 days, *p* = 0.02), and ICU length of stay (mean 4.59 vs. 1.27 days, *p* = 0.002) all exceeded those in the “post-PBC” group. As expected, timing metrics separated sharply: the mean time-to-change was 8.69 vs. 35.86 h, with changes ≤24 h (95.7% vs. 11.8%) and 24–72 h (3.2% vs. 30.1%) showing marked, statistically reliable differences (*p* < 0.001 for both). The “pre-PBC” group also showed a strong tendency toward broad-spectrum selection (79.8%) and almost all antibiotic therapies were remodified in the ICU (97%). Finally, appropriateness of therapy—assessed at 24, 24–48, and 48–72 h after the culture report—was higher in patients whose antibiotics had already been changed before a PBC (88.3%, 95.7%, and 95.7%, respectively), with differences that were highly robust using conventional methods (*p* < 0.001 for all; Table 4).

## 3. Discussion

We conducted a retrospective cohort study of 343 PBCs in the ED, comparing conventional organism identification with MALDI-TOF MS-based identification. In our once-daily workflow without active real-time antimicrobial stewardship oversight, MALDI-TOF MS implementation was not associated with differences in antibiotic appropriateness or in the timing and pattern of antibiotic modifications during the early hospitalization period. In a secondary analysis stratified by the timing of the first antibiotic change, patients whose antibiotics were modified before blood-culture positivity experienced higher mortality and a longer hospital length of stay, despite higher subsequent rates of appropriate antibiotic therapy.

Rates of appropriate empiric therapy vary across regions and healthcare settings and may differ substantially between general inpatient populations and emergency department cohorts. Systematic reviews have reported appropriateness rates of 50–90% for specific pathogens, highlighting resistance as a major driver of inadequacy in European and American hospitalized PBCs [6,18]. However, studies from East Asian healthcare institutions have documented high antimicrobial resistance in bloodstream isolates, which may result in lower AEA and higher mortality rates, suggesting the need for strong empiric guidelines and antimicrobial stewardship (AMS) implementation [19,20]. The severity of antimicrobial susceptibility profiles among common pathogens at CSMUH can be characterized as follows: among Gram-negative organisms, *Escherichia coli*, *Klebsiella pneumoniae*, *Pseudomonas aeruginosa*, and *Acinetobacter baumannii* are the four most frequently isolated pathogens, consistent with the predominant infection sources in our cohort—the urinary tract and gastrointestinal tract. Notably, the proportion of isolates classified as multidrug-resistant organisms and the complete resistance rate ranged from 27.7% to 42% across these Gram-negative and Gram-positive species. Consistent with this high-antimicrobial-resistance context, our 2018 institutional antibiogram, presented in the Supplementary Materials, shows reduced susceptibility among key Gram-negative pathogens to commonly used empiric agents, with particularly limited options in carbapenem-resistant phenotypes (e.g., *CRAB*, *CRPA*, and *CRE*). This epidemiologic backdrop helps explain why species-level identification alone may not translate into earlier optimization when susceptibility data are not accelerated.

Delays in appropriate antimicrobial treatment have been associated with increased 30-day mortality when effective treatment is not achieved within 12 h of blood-culture collection in patients with BSI, indicating a benchmark for providing rapid microbiological diagnostics of blood cultures [4,21]. In addition, the use of rapid diagnostic tests including MALDI-TOF MS plus real-time AMS may lead to a greater survival benefit than rapid tests or AMS alone [15]. However, variations in MALDI-TOF MS implementation —including routine colony-based identification in standard daily workflows, direct identification from positive blood-culture bottles, and continuous 24/7 operation—may contribute to heterogeneity in clinical outcomes such as mortality, length of stay, and rates of appropriate empiric antibiotic therapy [12,22,23]. In addition, cost-effectiveness and laboratory conversion to full automation should be considered in conjunction with potential clinical benefits. The RAPIDO trial economic evaluation found that adjunctive MALDI-TOF (in addition to conventional identification) was unlikely to be cost-effective based on its primary metric of incremental costs vs. 28-day survival [22,24,25]. At CSMUH, MALDI-TOF MS was performed on around 150 specimens collected the day before and operated in a once-daily batch workflow, which is less frequent than other published implementations but reasonable given the number of blood cultures obtained in the ED. This limited operational cadence may partly explain the absence of significant differences in appropriate empiric antibiotic use and in the timing of the first antibiotic modification in our study [26,27].

Virtually no prospective studies have examined evidence-based criteria for modifying empiric antibiotic therapy in the pre-PBC period in the ED, representing a critical “therapeutic window” where clinicians make decisions with maximum uncertainty. Research extensively documents that the time to blood-culture positivity varies considerably by pathogen and host factors, with approximately 70% detected within 24 h of incubation [28,29]. Time to positivity may also justify an earlier “antibiotic time-out”; one study estimated a residual probability of bacteremia of around 1.8% when cultures remain negative for >24 h, suggesting probable pretreatment [30]. In our study, the mean time to antibiotic modification before a PBC was 8.6 h, and 95% of modifications occurred within 24 h, despite an AEA rate as high as 88%, often with escalation but with worse LOS and mortality. This is exactly the pattern expected when clinicians escalate early in higher-acuity patients, reflecting confounding by indication, whereby clinicians are more likely to escalate or broaden therapy early in patients with greater physiologic derangement or perceived risk of deterioration [31,32]. In this context, “early modification” functions primarily as a marker of baseline severity rather than an independent causal determinant of outcome. Accordingly, our findings should be interpreted as reflecting a higher-acuity case-mix in the pre-positivity modification subgroup, and causal claims regarding the benefit or harm of earlier modification should be avoided without designs that address time-varying confounding [33,34]. However, modification of empiric therapy has been reported to be independently protective for in-hospital mortality in the ED setting, regardless of whether it occurs before or after a PBC, with a value comparable to infectious diseases specialist consultation [35,36].

Rapid diagnostic tests plus antimicrobial stewardship have been associated with improved outcomes and signals of survival benefit compared with conventional pathways [15]. However, diagnostic interventions may show little incremental antibiotic impact because of a ceiling effect when baseline stewardship is already strong [37]. In this context, using direct-from-positive-culture or short-incubation protocols to bring organism identification forward by 1 day may be beneficial for triggering reflex stewardship actions, such as SMS notifications based on local antimicrobial resistance patterns [15]. In our study, MALDI-TOF MS organism identification results were released in the HIS and were automatically displayed to clinicians with an on-screen alert and SMS notification to the ordering/responsible physician. However, there was no standardized, second-level antimicrobial stewardship intervention linked to MALDI-TOF MS reporting. Specifically, MALDI-TOF MS results were not routinely reviewed in real time by infectious diseases specialists or stewardship pharmacists, and no protocolized recommendation pathway was implemented. Overall, our neutral findings likely reflect an implementation model in which MALDI-TOF MS was performed once daily and results were delivered primarily through passive notification in high-antimicrobial-resistance settings. Early species identification alone may be insufficient to change a prescription when resistance markers and rapid phenotypic susceptibility data remain unavailable and the final AST turnaround time is unchanged. From a stewardship and public health perspective, high baseline antimicrobial resistance combined with frequent early broad-spectrum escalation underscores the need for systems that couple rapid diagnostics with actionable stewardship responses. Without such linkage, laboratory advances may have limited downstream impact, while antimicrobial selection pressure may remain substantial.

Our study has some limitations. First, its retrospective single-center design in a high- antimicrobial-resistance setting limits generalizability. Under these conditions, any reduction in time to blood-culture positivity processing and earlier organism identification may translate into limited clinical benefit, because the ultimate determination of the AEA is largely contingent on final antimicrobial susceptibility testing (AST), which was not accelerated by MALDI-TOF MS alone. Second, antimicrobial stewardship exposure was not directly measured. We were unable to directly quantify antimicrobial stewardship actions linked to MALDI-TOF MS results. Although preliminary reports were available through the HIS and automated notifications (alerts/SMS), the study did not capture a standardized real-time AMS intervention (e.g., pharmacist/ID review, documented recommendations, acceptance rates, or time-stamped therapy-change triggers). In addition, while telephone communication to responsible clinicians was routinely performed, these calls were not systematically documented. Third, residual confounding and unmeasured clinical/behavioral factors may remain. As an observational cohort, our analysis may be affected by residual confounding from factors not fully captured in the dataset, including invasive procedures and source-control interventions, differential ICU versus ward admission trajectories, and clinician-level prescribing variability. Prospective multicenter interventional trials are needed to test whether MALDI-TOF MS can increase AEA rates and improve patient outcomes in ED-onset PBCs. Fourth, cost-effectiveness was not formally measured, including laboratory and hospital staff aspects. Reviews of rapid diagnostics suggest that molecular rapid diagnostic test strategies paired with an ASP tend to be the most cost-effective approaches, compared with pure laboratory cost savings or MALDI-TOF MS alone [24,27,38].

## 4. Materials and Methods

### 4.1. Study Design and Setting

We conducted a before–after cohort study comparing positive blood cultures obtained before and after implementation of matrix-assisted laser desorption/ionization time-of-flight mass spectrometry (MALDI-TOF MS) (Vitek MS system, bioMerieux, Marcy-l’Étoile, France) in the Department of Laboratory Medicine at Chung Shan Medical University Hospital (CSMUH), a tertiary medical center in central Taiwan.

### 4.2. Study Period and Laboratory Methods

Data were collected from 1 May to 30 November 2018. Although MALDI-TOF MS went live in the microbiology laboratory on 13 August 2018, blood-culture reports from 1 to 31 August 2018 were excluded a priori because installation, relocation of legacy instruments, staff training, and early software/hardware stabilization could introduce a non-ignorable measurement error.

The MALDI-TOF MS platform used in our laboratory was the VITEK MS system (bioMérieux, Marcy-l’Étoile, France). In brief, bacterial colonies from positive cultures were applied to a target plate; after the matrix solution evaporated and crystallized, the crystals were irradiated with a laser. The matrix molecules absorbed energy and facilitated desorption and ionization of analytes. The resulting ions were accelerated through an electric field and analyzed by a time-of-flight mass analyzer, which records ion flight times and detects predominantly protein profiles, generating spectra characteristic of specific microorganisms. These spectra were matched against a reference database to identify organisms and to account for within-species diversity (with database coverage averaging >12 isolates per species and approximately 35 spectra per species). Each run could process up to 192 microbial samples. Results were reported using a simplified indicator system and organism name. Indeterminate or failed identifications were documented in detail and underwent subsequent review to determine potential causes.

All blood-culture processing followed the standard operating procedure of the CSMUH Department of Laboratory Medicine (SOP No. CSHL-SIP(BAC)-050501-HF0105). Bottles were incubated continuously in the BD BACTEC system (BD Diagnostics, Franklin Lakes, NJ, USA). When the instrument signaled a positive bottle, which occurred within <24 h in ≥95% of specimens, bottles were retrieved during staffed hours (08:00–17:00, Monday–Sunday) for immediate work-up with Gram stain. A preliminary microbiology report was issued based on Gram-stain morphology. Report timestamps were recorded in the electronic hospital information system (HIS), which also generated automated SMS notifications to the ordering clinician and displayed an on-screen alert within the HIS. During the pre-implementation window (1 May–31 July 2018), bacterial identification was performed using the Phoenix System (Becton Dickinson, Sparks, MD, USA). During the post-implementation window (1 September–30 November 2018), all identifications were performed using MALDI-TOF MS (VITEK MS system, bioMérieux, France). After a blood culture signaled positivity, the specimen was subcultured onto routine media and incubated to obtain isolated colonies. For clinically significant isolates, antimicrobial susceptibility testing (AST) was performed using a pure, fresh colony. Colonies were suspended in sterile saline and turbidity was standardized to the manufacturer-recommended 0.5 McFarland (or equivalent) using a turbidity meter. The standardized suspension was loaded into the automated AST platform (VITEK-2, bioMérieux) with organism-appropriate identification and AST cards. The instrument performed automated inoculation, incubation, and optical monitoring of bacterial growth in the presence of graded antibiotic concentrations to generate minimum inhibitory concentrations (MICs). MICs were interpreted as susceptible/intermediate/resistant according to CLSI 2018 [39] breakpoints and laboratory rules. Quality control procedures were performed in accordance with laboratory policy, using standard reference strains to verify the accuracy of MIC results and system performance. AST results were released as the final blood-culture report in the hospital information system, with automated clinician notification per institutional protocol.

### 4.3. Inclusion and Exclusion Criteria

Inclusion criteria were ED patients with positive blood cultures, for whom blood samples were obtained in the ED during the study windows described above. In our study, an infectious episode (a single positive blood-culture event) was defined using positive aerobic, anaerobic, and pediatric blood-culture bottles. All blood-culture bottles were routinely incubated for up to 5 days, which is the standard observation period to allow organism growth and detection before cultures are finalized as negative. Exclusion criteria included the following: (1) Common skin contaminants, as defined by the CSMUH microbiology laboratory policy: For adult blood cultures, growth of only one set with any of the following organisms was classified as contamination and not subjected to antimicrobial susceptibility testing (AST): coagulase-negative *Staphylococcus*, *Micrococcus* spp., *Bacillus* spp., *Corynebacterium* spp., *Propionibacterium* spp., or *Aerococcus* spp. If any of these organisms grew in two or more sets or if the treating physician requested a work-up, they were treated as clinically significant pathogens and underwent full identification and AST. For pediatric blood cultures, coagulase-negative *Staphylococcus* or alpha-*hemolytic Streptococcus* species were considered clinically significant and were fully worked up. (2) Duplicate positive cultures from the same infectious episode, defined as recovery of the same pathogen from the same patient within three days of the initial blood-culture collection: Such cultures were treated as a single infectious episode for analysis.

Baseline variables included basic characteristics (age and sex), key medical history (malignancy, ventilator dependence, cerebrovascular disease, maintenance hemodialysis, and a do-not-resuscitate order or palliative/hospice status), and clinical status variables, including presumed source of infection, septic shock during the ED observation period, ED triage category, qSOFA score, time of the initial positive blood culture (PBC) report (Gram stain), and time of the final report (organism identification with antimicrobial susceptibility testing).

Clinical outcome measures included hospital and ICU LOS, in-hospital mortality, appropriate antibiotic use within 24, 48, and 72 h after the first PBC report, and the time, location, and classification of the first antibiotic modification. Antibiotic therapy was deemed appropriate if the regimen was active in vitro against the final blood-culture pathogens per AST, with a guideline-concordant dose and route, accounting for renal/hepatic function. For polymicrobial bacteremia, activity against the composite pathogen profile was required. All data were extracted from the CSMUH electronic hospital information system by an emergency physician, with supplemental aggregation from microbiology laboratory blood-culture statistics to characterize ED management of patients with suspected infection.

### 4.4. Hypothesis and Endpoints

We hypothesized that earlier organism identification via MALDI-TOF MS, implemented as once-daily batch reporting with passive notification, would translate into more appropriate antibiotic selection and modification throughout the earlier admission course. The primary endpoints were the proportions of patients receiving appropriate antibiotics at 24, 48, and 72 h after the blood culture became positive in the laboratory. The secondary endpoint was the pattern of the first antibiotic change (timing, care location, and escalation vs. de-escalation classification).

### 4.5. Statistical Analysis

Continuous variables were expressed as median and interquartile range (IQR), and categorical variables were expressed as counts and percentages. Comparisons between groups were performed using the chi-square test or Fisher’s exact test for categorical variables and the Mann–Whitney U test for continuous variables. A *p*-value < 0.05 was considered statistically significant, and statistical analyses were performed using SPSS version 18.0.

## 5. Conclusions

In this high-antimicrobial-resistance cohort with positive blood cultures obtained in the emergency department, implementation of once-daily MALDI-TOF MS—without active real-time stewardship oversight—was not associated with improved early antibiotic appropriateness, antibiotic modification patterns, or in-hospital mortality compared with conventional identification. Future prospective studies should evaluate whether higher-frequency or 24/7 processing, direct-from-positive-bottle identification, and integrated real-time antimicrobial stewardship can better translate faster microbiologic identification into timely therapeutic action.

## Figures and Tables

**Table 1:** Emergency department patient characteristics with positive blood culture.

		Conventional Method N = 182	MALDI-TOF Method N = 141	*p* Value
First report time (hours)				
	Mean number	26.05	27.35	0.583
	Median number	20 (IQR: 16~26)	21 (IQR: 16~28)	0.227
Final report time (hours)				
Mean number		81.46	82.46	0.844
Median number		69 (IQR: 60~88)	69 (IQR: 61.5~86.5)	0.815
Mortality		27 (14.8%)	21 (14.9%)	0.988
Disposition	ICU	33 (18.1)	25 (17.7)	0.763
	Ward	121 (66.5)	98 (69.5)	
	Discharge	28 (15.4)	18 (12.8)	
ICU stay (days)		2.2	2.28	0.920
Ward (days)		11.11	13.55	0.078
Age (years)		70.6	67.7	0.060
Sex (male)		87 (47.8)	71 (50.4)	0.655
DNR		11 (6)	6 (4.3)	0.617
Hemodialysis		9 (4.9)	11 (7.8)	0.354
Malignancy		45 (24.7)	43 (30.5)	0.259
CVA		22 (12.1)	9 (6.4)	0.090
Endotracheal intubation		7 (3.8)	3 (2.1)	0.522
qSOFA ≥ 2		27 (14.8)	13 (9.2)	0.173
Septic shock		48 (26.4)	26 (48.4)	0.190
Infection source	Urinary tract	66 (36.3)	52 (36.9)	0.782
	Gastrointestinal	42 (23.1)	34 (24.1)	
	Pulmonary	18 (9.9)	14 (9.9)	
	Catheter-related	3 (1.6)	2 (1.4)	
	Mix—infection	13 (7.1)	12 (8.5)	
	Other	30 (16.5)	15 (10.6)	
	Soft tissue	10 (5.4)	12 (8.5)	

ICU: Intensive Care Unit; CVA: Cerebrovascular Accident; DNR: Do Not Resuscitate.

**Table 2:** Appropriate antibiotics rate and type of switch.

		Conventional Method N = 182 (%)	MALDI-TOF Method N = 141(%)	*p* Value
Abx switch after B/C positive <24 h	Unchanged	141 (77.5)	100 (70.9)	
	Broader spectrum	35 (19.2)	35 (24.8)	0.407
	Narrow spectrum	6 (3.3)	6 (4.3)	
Abx switch after B/C positive 24–48 h	Unchanged	158 (86.8)	121 (85.8)	
	Broader spectrum	13 (7.1)	15 (10.6)	0.345
	Narrow spectrum	11 (6.0)	5 (3.5)	
Abx switch after B/C positive 48–72 h	Unchanged	148 (81.3)	124 (87.9)	
	Broader spectrum	16 (8.7)	9 (6.4)	0.401
	Narrow spectrum	18 (9.9)	8 (5.7)	
Appropriate Abx after B/C positive	<24 h	137 (75.3)	108 (76.6)	0.444
	24–48 h	150 (82.4)	113 (80.1)	0.666
	8–72 h	157 (86.3)	119 (84.4)	0.751

Abx: Antibiotics; B/C: Blood Culture.

**Table 3:** Analysis of first antibiotic therapy switch.

		Conventional Method N = 182 (%)	MALDI-TOF Method N = 141(%)	*p* Value
Abx first switch time (hour)	Mean number	25.46	31.16	0.206
	Median number	6 (IQR: 0~48)	15 (IQR: 0~40.5)	0.090
Abx switch category	Unchanged	57 (31.3)	36 (25.5)	0.316
	<24 h	67 (36.8)	50 (35.5)	
	24–72 h	40 (22.0)	32 (22.7)	
	>72 h	18 (9.9)	23 (16.3)	
	Unchanged	58 (31.9)	36 (25.5)	0.451
Abx first switch type	Broader spectrum	91 (50)	76 (53.9)	
	Narrow spectrum	33 (18.1)	29 (20.6)	
	Unchanged	57 (31.3)	36 (25.5)	0.164
	ICU	25 (13.7)	23 (16.3)	
Abx first switch location	Ward	82 (45.1)	76 (53.9)	
	OPD	3 (1.6)	2 (1.4)	
	ED	15 (8.2)	4 (2.8)	
Abx switch before B/C positive		59 (32.4)	35 (24.8)	0.141

Abx: Antibiotics; ICU: Intensive Care Unit; ED: Emergency Department; OPD: Outpatient Department; B/C: Blood Culture.

**Table 4:** Analysis of switch before and after blood culture showed positive results.

		Abx Modification After B/C Positive N = 229 (%)	Abx Modification Before B/C Positive N = 94 (%)	*p* Value
Abx first switch time (hours)	Mean number	35.86	8.69	<0.001
ICU stay (day)		1.27	4.59	0.002
Ward stay (day)		11.17	14.63	0.022
Abx switch category	Unchanged	93 (40.6)	0 (0)	<0.001
	<24 h	27 (11.8)	90 (95.7)	
	24–72 h	69 (30.1)	3 (3.2)	
	>72 h	40 (17.5)	1 (1.1)	
	Unchanged	93 (40.6)	0 (0)	<0.001
Abx first switch type	Broader spectrum	93 (40.6)	75 (79.8)	
	Narrow spectrum	43 (18.8)	19 (20.2)	
	Unchanged	93 (40.6)	0 (0)	<0.001
	ICU	15 (6.6)	33 (35.1)	
Abx first switch location	Ward	109 (47.6)	49 (52.1)	
	OPD	5 (2.2)	0 (0)	
	ED	7 (3.1)	12 (12.8)	
qSOFA ≥ 2		19 (8.3)	21 (22.3)	0.001
Mortality		28 (12.2)	20 (21.3)	0.041
Disposition	ICU Ward Discharge	24(10.5) 159 (69.4) 46 (20.1)	34 (36.2) 60 (63.8) 0 (0)	<0.001
Septic shock		38 (16.6)	36 (38.3)	<0.001
Appropriate Abx after B/C positive	<24 h 24–48 h 48–72 h	162 (70.7) 173 (75.5) 186 (81.2)	83 (88.3) 90 (95.7) 90 (95.7)	0.001 <0.001 0.001

Abx: Antibiotics; ICU: Intensive Care Unit; ED: Emergency Department; B/C: Blood Culture; OPD: Outpatient Department.

## Data Availability

The data presented in this study are available on request from the corresponding authors upon request.

## Supplementary Materials

The following supporting information can be downloaded at: https://www.mdpi.com/article/10.3390/antibiotics15040386/s1, Supplementary Data: Antimicrobial susceptibility patterns of common pathogens.
