# Supplementary material for: Association of Once-Daily MALDI-TOF MS Identification with Antibiotic Appropriateness and First-Modification Patterns in Emergency Department Bacteremia: A Retrospective Before–After Cohort Study
Source: Antibiotics (Basel). 2026 Apr 10;15(4):386. doi: 10.3390/antibiotics15040386 (PMC13113376; doi:10.3390/antibiotics15040386)
Supplement: Supplementary file 1 [file antibiotics-15-00386-s001.zip › antibiotics-4199050-supplementary.pdf]

antibiotics

Chung Shan Medical University Hospital: Antimicrobial Susceptibility Patterns of Common pathogens

Gram-Positive Pathogens (2018)

	VA	P	OX	E	CC	LZD	TE	CIP	LVX	AM	GMS	SXT	DAP	CTX	FEP	MFX	FA
Staphylococcus aureus(425)	100	7	60	51	71	100	59	78	78	-	-	91	100	-	-	-	94
Staphylococcus aureus(MRSA)(185)	100	0	0	12	38	100	47	37	-	-	-	73	100	-	-	-	89
Staphylococcus epidermidis(31)	100	0	7	33	50	100	70	53	57	-	-	52	100	-	-	-	48
Streptococcus agalactiae(110)	100	100	-	59	57	100	-	-	95	-	-	-	-	100	100	-	-
Streptococcus pyogenes(4)	100	100	-	75	75	100	-	-	100	-	-	-	-	100	100	-	-
Streptococcus pneumoniae(14)	100	57	-	14	14	100	21	-	93	-	-	79	-	77	-	93	-
Enterococcus faecalis(242)	99	99	-	-	-	99	-	61	-	99	57	-	100	-	-	-	-
Enterococcus faecalis(VREfs)(0)	-	-	-	-	-	-	-	-	-	-	-	-	-	-	-	-	-
Enterococcus faecium(213)	50	8	-	-	-	99	-	5	-	9	42	-	100	-	-	-	-
Enterococcus faecium(VREfm)(53)	0	0	-	-	-	99	-	0	-	0	22	-	100	-	-	-	-

VA Vancomycin   P Penicillin   OX Oxacillin   E Erythromycin

CC Clindamycin   LZD Linezolid   TE Tetracycline   CIP Ciprofloxacin

LVX Levofloxacin   AM Ampicillin   GMS Gentamicin-Synergy

SXT Trimethoprim-Sulphamethoxazole   DAP Daptomycin   CTX Cefotaxime

FEP Cefepime   MFX Moxifloxacin   FA Fusidic Acid

https://doi.org/10.3390/antibiotics15040386

Chung Shan Medical University Hospital: Antimicrobial Susceptibility Patterns of Common pathogens

Gram-Negative Pathogens (2018)

	AN	GM	AM	TZP	SAM	CZ	CAZ	CRO	FEP	ETP	MEM	IPM	CIP	LVX	SXT	CXM
Escherichia coli(1249)	99	77	23	92	38	54	73	67	76	98	100	98	59	57	54	-
Escherichia coli(CREC)(11)	89	37	0	11	0	0	11	11	16	11	80	16	26	27	37	-
Klebsiella pneumoniae(626)	97	77	0	83	62	55	75	77	84	88	93	84	83	84	70	-
Klebsiella pneumonia(CRKP)(65)	80	26	0	7	0	0	1	6	16	7	20	7	23	16	26	-
Klebsiella aerogenes(53)	98	87	0	68	6	0	66	68	93	91	97	74	81	87	83	-
Klebsiella oxytoca(36)	100	94	0	97	53	14	86	83	89	94	100	92	94	89	94	-
Enterobacter cloacae(83)	99	90	0	74	7	0	68	64	87	80	100	89	95	94	83	-
Enterobacter cloacae(CRECL)(5)	83	83	0	17	0	0	0	0	17	0	0	33	83	0	50	-
Proteus mirabilis(178)	96	69	45	99	70	49	94	91	94	100	99	49	70	64	49	-
Citrobacter freundii(13)	100	92	0	92	23	0	77	77	92	92	100	77	92	100	85	-
Citrobacter koseri(70)	99	97	0	100	90	84	97	96	99	100	100	100	100	100	96	-
Serratia marcescens(77)	99	90	0	86	3	0	94	79	92	97	100	55	83	77	94	-
Morganella morganii(56)	100	73	0	95	20	0	91	96	100	100	100	43	80	75	64	-
Aeromonas hydrophila(14)	100	71	-	64	-	-	100	79	93	14	50	64	93	83	86	-
Salmonella enteritidis(68)	-	-	52	-	-	-	86	94	-	-	-	-	68	100	71	-
Haemophilus influenzae (39)	-	-	31	-	51	-	-	100	-	-	92	-	-	-	44	85

Glucose nonfermentative bacteria

	TGC	AN	GM	TZP	SAM	MEM	IPM	CAZ	CRO	FEP	CIP	LVX	SXT	CO
Acinetobacter baumannii(274)	84	79	52	58	75	64	64	70	50	73	68	73	62	99
Acinetobacter baumannii(CRAB)(117)	70	48	18	1	42	-	0	22	16	42	23	-	5	97
Pseudomonas aeruginosa(487)	-	98	92	83	-	85	87	87	-	89	85	79	-	99
Pseudomonas aeruginosa(CRPA)(49)	-	89	78	46	-	-	0	62	-	70	62	-	-	98
Stenotrophomonas maltophilia(178)	85	-	-	-	-	-	-	38	-	-	-	79	95	-

AN Amikacin **GM** Gentamicin **AM** Ampicillin **TZP** Piperacillin/Tazobactam

SAM Ampicillin/Sulbactam **ETP** Ertapenem **MEM** Meropenem **IPM** Imipenem **CZ** Cefazolin

CAZ Ceftazidime **CRO** Ceftriaxone **FEP** Cefepime **CIP** Ciprofloxacin **LVX** Levofloxacin

SXT Trimethoprim-Sulphamethoxazole **CXM** Cefuroxime **TGC** Tigecycline **CO** Colistin

https://doi.org/10.3390/antibiotics15040386
